# Deciphering the potential role of Maca compounds prescription influencing gut microbiota in the management of exercise-induced fatigue by integrative genomic analysis

**DOI:** 10.3389/fnut.2022.1004174

**Published:** 2022-10-12

**Authors:** Hongkang Zhu, Ruoyong Wang, Hanyi Hua, He Qian, Peng Du

**Affiliations:** ^1^State Key Laboratory of Food Science and Technology, Jiangnan University, Wuxi, Jiangsu, China; ^2^Collaborative Innovation Center of Food Safety and Quality Control in Jiangsu Province, Jiangnan University, Wuxi, Jiangsu, China; ^3^School of Food Science and Technology, Jiangnan University, Wuxi, Jiangsu, China; ^4^Air Force Medical Center, Beijing, China

**Keywords:** anti-fatigue, gut microbiota, microbial function, oxidative stress, host metabolism

## Abstract

A growing number of nutraceuticals and cosmeceuticals have been utilized for millennia as anti-fatigue supplements in folk medicine. However, the anti-fatigue mechanism underlying is still far from being clearly explained. The aim of the study is to explore the underlying mechanism of the Maca compound preparation (MCP), a prescription for management of exercise-induced fatigue. In this study, mice weight-loaded swimming test was used to evaluate the anti-fatigue effect of MCP. MCP significantly improved the forelimb grip strength and Rota-rod test in behavioral tests *via* regulating energy metabolism. 16S rDNA sequencing results showed MCP can regulate the intestinal flora at the genus level by increasing several beneficial bacteria (i.e., *Lactobacillus*, *Akkermansia* and etc.), and decreasing the harmful bacteria (i.e., *Candidatus_Planktophila* and *Candidatus_Arthromitus*), where notable high relevance was observed between the fatigue-related biomarkers and fecal microbiota. The results of microbial function analysis suggested that MCP might improve exercise-induced fatigue by enhancing energy metabolism, carbohydrate and lipid metabolism and metabolism of terpenoids and polyketides and breakdown of amino acid metabolism. In addition, and H_2_O_2_-induced oxidative stress model on C2C12 cells was employed to further validate the regulation of MCP on energy metabolisms. MCP pre-treatment significantly reduced intracellular ROS accumulation, and increased glycogen content, ATP generation capacity and mitochondrial membrane potential of skeletal muscle cells, as well as conferred anti-cell necrosis ability. In conclusion, MCP plays a key role in regulating fatigue occurrence in exercising and gut microbiota balance, which may be of particular importance in the case of manual workers or sub-healthy populations.

## Introduction

Fatigue is a systemic process in the decline of human physiology and psychology, and most of the homeopathic supplements are holistic treatments. A growing number of edible natural plants have become used in dietary supplements for relieving fatigue-related symptoms (1, 2), where the compound prescriptions has become one of the most commonly used forms for exercise fatigue (3). Maca compound preparation (MCP) has been administered as an anti-fatigue agent in folk medicine in China for a long time according to *The Medical Classic of the Yellow Emperor* and *Compendium of Materia Medica*. In this formula, Maca (*Lepidium meyenii* Walp.), the monarch drug, is an edible medicine and a new resource food and its anti-fatigue effect has been investigated in our previous study (4). Macamides are the major constituents in Maca and their anti-fatigue activity is mainly credited to their antioxidant and anti-inflammatory properties (5). The eight edible and medicinal plants in MCP (Table 1) have been used to treat fatigue or weakness, which can also inhibit oxidative stress or inflammatory injury induced by exhausting physical exercise.

**TABLE 1:** The list of the edible or medicinal plants in Maca compound preparation (MCP).

No.	Latin name	Purity	Used part	Traditional use	Representative active ingredients	References
1	*Lepidium meyenii* Walp.	30%	Root	Anti-fatigue, improving sexual functions	Macamides and macanes	(4, 5)
2	*Polygonatum sibiricum*	10%	Root	Anti-aging, anti-diabetic, anti-fatigue, and anticancer	DFV, baicalein, 3′-Methoxydaidzein, beta-sitosterol, sitosterol, methylprotodioscin_qt, (2R)-7-hydroxy-2-(4-hydroxyphenyl)chroman-4-one, diosgenin, 4′,5-Dihydroxyflavone, sibiricoside A	(11)
3	*Astragalus membranaceus*	10%	Root	Immune stimulant, tonic	chrysanthemaxanthin, cnidilin, folinicacid, isoimperatorin, phyllanthin, suchilactone, beta-sitosterol, stigmasterol	(12)
4	*Citrus reticulata Blanco*	10%	Peel	Anti-fungal, refreshing	carotene, citromitin, nobiletin, obacunone, β-sitosterol, sitosterol, 5,7-dihydroxy-2-(3-hydroxy-4-methoxyphenyl)chroman-4-one, naringenin	(13)
5	*Codonopsis pilosula*	10%	Root	Improving immune, blood, digestive system	5α-stigmastan-3,6-dione, alpha-spinasterol, chrysanthemaxanthin, daturilin, diisocapryl phthalate, friedelin, frutinone, glycitein,	(14)
6	*Polygonatum odoratum* (Mill.) Druce	10%	Root	Antidiabetic, reduce blood glucose, hypolipedemic	Isoflavanone, 7-O-methylisomucronulatol, formononetin, FA, Mairin, 3,9-di-O-methylnissolin, Jaranol, isorhamnetin, isomucronulatol-7,2′-di-O-glucosiole, Calycosin, quercetin, kaempferol, 5′-hydroxyiso-muronulatol-2′,5′-di-O-glucoside	(15)
7	*Angelica sinensis*	10%	Root	Regulating menstruation and removing pain	meletin, quercetin, stigmasterol, 24-Ethylcholest-4-en-3-one, poriferast-5-en-3beta-ol, Sitosteryl acetate, beta-sitosterol, vitamin-e	(16)
8	*Amomum villosum Lour.*	10%	Seed	Removing damp-cold in the spleen	4′,5,7-trihydroxy-6,8-dimethyl-homoisoflavanone, polygosides, 4′,5,7-trihydroxy-6-methyl-homoisoflavanone, 4′-methoxy-5,7-dihydroxy-6,8-dimethyl-homoisflavanone, n-coumaroyltyramine	(17)

With the in-depth study of intestinal flora, gut microbiota derived metabolites have been confirmed closely related to the progression of bidirectional communication pathways linking to host (6, 7). Therefore, the overall treatment targeting on both energy metabolism and intestinal bacteria may be a worthwhile therapeutic strategy to fatigue management.

In our previous study, the active components of MCP and anti-fatigue potential have been predicted by network pharmacology exploration (8). However, the effect of MCP on anti-fatigue has not been fully explained and validated, and meanwhile, the association between fatigue-related effects and microbial function has not been established yet. In this study, the weight-loaded forced swimming test (WFST) was performed to verify its anti-fatigue effect, and the underlying mechanisms were systematically explored in exercise-induced fatigue mice. To further explore probable associations between its anti-fatigue effects and gut prebiotic capacity, we tried to measure some preliminary link between physical fatigue and gut microbiota in genus-level. The PICRUSt2 algorithm and MicroCyc databases (9, 10) were employed to investigate the metabolic functions of gut microbes and how MCP regulated gut microbiota and metabolic processes in the fatigue. Based on Kyoto Encyclopedia of Genes and Genomes pathway (KEEG) pathways analysis, metabolism pathways at level 1 are critically involved in host-microbial interactions, where energy metabolisms were significantly promoted by MCP. Finally, a typical H_2_O_2_-induced C2C12 cell injury model was applied to mimic skeletal muscle oxidative injury to investigate the protective effects of antioxidant pretreatment and validated the regulations of the intracellular energy metabolisms by MCP *in vitro*.

## Materials and methods

### Preparation of Maca compound preparation

*Lepidium meyenii* Walp. was planted on the Tibet Plateau with an altitude over 3,000 meters. It was harvested in December, and the other 7 plants in MCP were harvested in Shandong Province, China, and were positively identified as the species by Guoliang Ding (Registered Traditional Chinese Medicine Practitioner, R.TCM.P., China).

The main medicinal parts of plants in MCP (Table 1) were cut into thin sections (3–5 mm). After soaking for half an hour, MCP was extracted in boiling water (w/v, 1:8) twice for 1 h at atmospheric pressure. After filtration, the hot water extracts were combined and cooled to room temperature prior to centrifuge at 3,000 rpm for 10 min, further removing the suspended residue in the filtrate. MCP is of high nutritional value, containing 34.78 ± 2.43 mg/ml of total polysaccharides, 0.157 ± 0.018 mg/ml of flavonoids and 1845.27 ± 10.92 mg/ml of total amino acids according to the published paper which used the same protocol (8). The MCP extract was lyophilized at −70°C using a freeze dryer. All lyophilized extracts powder was sealed in sterile sampling bags and stored at −80°C for further experiments.

### Anti-fatigue effect of Maca compound preparation *in vivo*

#### Experimental animals

**FIGURE 1:**
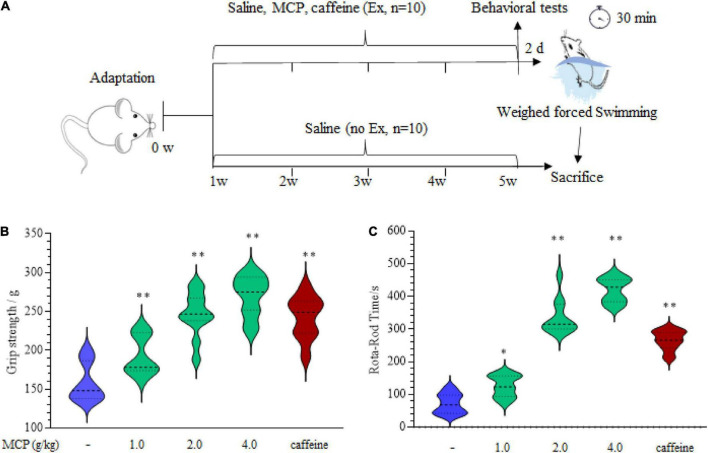
Effects of Maca compound preparation (MCP) on the mice physical fitness measurement tests. **(A)** Scheme of animal experiments of the weighed-forced swimming test (WFST, 5% bodyweight). **(B)** The forelimb grip strength of mice examined by grip-strength test. **(C)** The motor coordination of mice in Rota-rod test. *p < 0.05, ^**^p < 0.01 vs. control group (saline).

All experimental animal procedures were approved by the Ethics Committee of Experimental Animal Center of Jiangnan University (JN.No 20200710i0720915) and the whole project team was strongly committed to comply with the ethical policy. The experiments were performed when Institute of Cancer Research (ICR) mice (18–22 g, male) had adapted to the experimental environment for a week. Animals were randomly divided into 6 different groups (*n* = 10): control with vehicle treatment (Con); swimming exercise with vehicle treatment (Ex); swimming exercise with MCP (Ex + MCP) or caffeine (purity ≥ 99.8%, Pos, 10 mg/kg bw.) every day for 30 days. The vehicle group received the same volume of sterile water and the 1/10th of the recommended dosage for humans of MCP (12 g/day) received by the mice was based on the “mouse equivalent dose” (8, 18). Thus, MCP was orally administered at 1.0, 2.0, or 4.0 g/kg bw., respectively (the calculated process described in the Appendix), which was reconstituted in sterile water. The low, moderate, and high dose levels of MCP were labeled as MCP-L, MCP-M, MCP-H (Figure 1A).

#### Behavioral test

##### Grip strength test

The forelimb grip strength was assessed by a grip strength meter (YLS-13A Jinan Yiyan Technology Development Co., Ltd., Jinan, China) after oral gavage for 4 weeks. Each mouse was lifted by tail, so its forepaws could grip the wire of the strength meter, and then gently pulled back with tail parallel to table until the mouse lost its grip on the wire. Three tests were performed in succession on each mouse, and scores were averaged for statistical analysis.

##### Rota-rod test

Mice from each group were placed on an accelerating rota-rod cylinder (ZB-200, Chengdu Taimeng Science Technology Co., Ltd.). The rota-rod was accelerated from 5 to 15 rpm in 5 min. Each mouse remained on revolving rod for the training period. In the formal test, mice were placed on the rota-rod at speed of 15 rpm, until they were exhausted and dropped from the rod. The time of duration was recorded to evaluate motor coordination.

#### Weight-loaded forced swimming test

Weight-loaded forced swimming test (WFST) was carried out after orally administration on the last day (19, 20). The mice were placed in a tank (height: 65 cm; diameter: 40 cm) of room temperature water (25 ± 2°C) with a depth of 25 cm. The mice were loaded with a lead block (5% of body weight) attached to tails. After the WFST for 30 min, mice were sacrificed for the fatigue-related biochemical indexes measurement.

#### Biochemical indexes measurement

The blood was detected for blood sugar by Roche blood sugar meter (ACCU-CHEK Performa) and centrifugated at 3,500 *g* for 10 min at room temperature for measurement of blood lactic acid (BLA), blood urea nitrogen (BUN), lactate dehydrogenase activity (LDH) according to the instructions of the assay kit (Jiancheng Biotechnology Co., Nanjing, China). The left hind leg thigh muscle of mice was collected to measure the levels of reactive oxygen species (ROS), adenosine-triphosphate (ATP), nicotinamide adenine dinucleotide (reduce) [NAD(H)] and glycogen, which were detected (Fankew, Shanghai FANKEL Industrial Co., Ltd., Shanghai, China).

#### Hematoxylin and eosin staining

For histological examination, the muscle tissue was fixed in 4% (v/v) paraformaldehyde/PBS and embedded in paraffin, then stained the tissue with H&E. Finally, images were acquired from light microscopy (Olympus, Tokyo, Japan) (×200).

#### 16S rRNA gene and bioinformatics analysis

The 5 mice were selected from Ex and MCP-M groups at random, and DNA from feces was extracted by using a Genomic DNA Kit (Omega Bio-tek, Inc., Norcross, GA, USA). The 16S rRNA genes (V3-V4 regions) were amplified from the whole genome via the primer pair (341 F, 5′- CCTAYGGGRBGCASCAG-3′; 806R, 5′-GGACTACHVGGGTWTCTAAT-3′). All the amplicons were purified, quantified, and sequenced on an Illumina novaseq platform (San Diego, CA, USA). The barcode and connector sequence were removed. FLASH (v1.2.8) was used to stitch the double-ended sequences, and Vsearch (v2.3.4) was used to filter out the unqualified sequences (8). Finally, the sequence with 97% similarity was classified as an OTU. 16S rRNA gene reads were down-sampled to a read depth of 44,367 reads/sample and reads mapped to 16S OTUs (1,635 reads), to ensure sample compatibility regardless of sampling depth (21). Gene functions were predicted by PICRUSt.2 and analyzed by KEGG pathway enrichment analysis.^1^

### Effect of Maca compound preparation on cellular metabolism in C2C12 cells

#### Cell culture and differentiation

The C2C12 (mouse skeletal muscle) cells were purchased from (Hunan Fenghui Biotechnology Co., Ltd., Changsha, China). The cells were maintained in growth medium [Dulbecco’s modified Eagle medium (DMEM) supplemented, 10% (v/v) fetal bovine serum (FBS), 1% (v/v) Penicillin-Streptomycin for cell culture (*Gibico*)] in a 25 cm^2^ culture flask. The cells were cultivated at 37°C under a humidified atmosphere of 5% (v/v) CO_2_. To induce cell differentiation, 70% confluent cultures were switched to DMEM containing 2% horse serum (HS) and 10 μg/ml of insulin for 3 days with medium changes every other day.

#### H_2_O_2_-induced oxidative stress on C2C12 cells

When the C2C12 cells were grown to approximately 70–80% confluence in 96-well flat-bottomed plates (*n* = 5), they were replenished MCP (re-dissolved in basal medium, free of FBS), and incubated for a further 24 h. According to a previous study (4), cells were pre-treated with 0.48 mmol/L H_2_O_2_ for 6 h to inflict oxidative stress before harvest. The cell viability of reagents on C2C12 cells was, respectively, assessed using Cell Counting Kit-8 (CCK-8, *Beyotime*) assays. After incubating for 1h, the absorbance of each well was measured at 450 nm with a microplate reader (BioTek Instruments, Winooski, VT, USA).

#### Measurement of reactive oxygen species level in C2C12 cells

The levels of ROS in C2C12 cells were determined using fluorescent probe (DCFH-DA, *Beyotime*, China). For flow cytometry analysis, C2C12 cells were seeded in 6-well plates at a density of 5 × 10^5^ cells per well (treatment of cells was shown in 2.2.2). Both suspended and adherent cells were collected and washed with PBS twice (for washing out the trypsin). The cells were stained with DCFH-DA and incubated at dark ambient for 20 min. After re-suspending in PBS, they were analyzed by flow cytometry (FACSAriaII, Becton, Dickinson and Company, Franklin Lakes, NJ, USA) according to the manufacturer’s protocol. Analysis of flow cytometry data was performed with FlowJo software (v10.8.1).

#### Measurement of energy metabolism level in C2C12 cells

To evaluate the effect of the compounds on energy metabolism during muscle growth, the anthrone reagent was employed in the estimation of glycogen content by use of glycogen assay kit (Solarbio Science & Technology Co. Ltd., Beijing, China). C2C12 cells were seeded in 6-wells plates, and 5 million cells would be harvested in 2 days. The glycogen content in cells was measured with an anthrone color reagent from an alkaline digest in concentrated sulfuric acid and calculated in the number of 10^4^ cells.

ATP levels in C2C12 cells were measured by an enhanced ATP assay kit (Beyotime Inc., Shanghai, China) as described in manufacturer’s instructions. Luminescence in the supernatant from each sample was measured in a Synergy Mx multifunctional Microplate Reader (Gene Company Ltd., Hongkong, China). Data were normalized to the control as 100%.

#### Calcein-AM/PI double staining of C2C12 cells

The Calcein-AM/PI Double Stain Kit (Dojindo Laboratories, Kumamoto, Japan) was used to assess the live/dead staining assay. In brief, the C2C12 cells were seeded onto glass coverslips in 96-well cell culture plates. Cells were treated as shown in 2.2.2. After co-incubation with agents (for 24 h) and H_2_O_2_ (for 6 h), cells were washed twice with 1 × Assay Buffer, then loaded with Calcein-AM and Propidium Iodide (PI) at 37°C for 20 min, washed twice in staining buffer, and fluorescence were measured by inverted fluorescence microscope (Carl Zeiss, Jena, Germany). The experiments were performed on C2C12 cells from 3 different visions at random.

#### Measurement of mitochondrial membrane potential (MMP, ΔΨm)

Changes in ΔΨm of C2C12 cells were measured by using a mitochondrion-specific cationic dye JC-1 according to manufacturer’s instructions (*Beyotime*) (22). Cells were incubated with JC-1 working solution in the dark for 20 min, which was measured by high-resolution confocal laser microscope (LSM880, Carl Zeiss, Germany). Red-fluorescent emissions were formed by J-aggregates at high membrane potential (excitation 585 nm, emission 590 nm), whereas green-fluorescent monomers existed at low potential (excitation 514 nm, emission 529 nm). Mitochondrial depolarization manifests by a decrease in the ratio of the red and green fluorescence.

### Network pharmacology and molecular docking analysis

Swiss Target Prediction^2^ was used to predict all the targets of candidates, which were imported into Cytoscape 3.8.0 to construct a drug-target network. Molecular docking was carried out online by using SwissDock.^3^ Crystal structures of the four validated targets in PDB format were downloaded from AlphaFold Protein Structure Database^4^ and uploaded with candidate ligands in MOL2 format. The lowest Gibbs free energy (△G) and fullfitness of each interaction were calculated *in silico*.

### Data analysis

GraphPad Prism 9.0 was used for analysis. Results were expressed as mean ± standard deviation. One-way ANOVA with Dennett’s multiple comparisons test was used for comparison between the three groups, *p < 0.05, ^**^p < 0.01 were statistically significant among the groups. The correlation among these indicators was conducted by Pearson correlation analysis using R software version 4.1.0.

## Results and discussion

### Effect of Maca compound preparation on the mice physical fitness

The imbalance of metabolic utilization in peripheral muscle may be both the cause and result of exercise-induced fatigue (23). To validate the effect of MCP on the physical fitness, the grip-strength test and Rota-rod test were carried out after administration of MCP for 4 weeks, as shown in Figure 1A. There is a significant improvement in the forelimb grip strength (Figure 1B), demonstrating MCP enhanced mice muscle strength force production. Meanwhile, the difference seemed to be implicitly stated in Rota-rod exercising (Figure 1C), since motor abilities on accelerating Rota-rod increased significantly in MCP mice. These results revealed improved locomotor capacity in mice treated with MCP. However, to determine whether MCP acted as an antagonist on fatigue/tiredness produced by exercise, we employed the WFST model to investigate the effects of MCP on muscle fatigue status *in vivo*.

### Effect of Maca compound preparation on mice energetic metabolism

**FIGURE 2:**
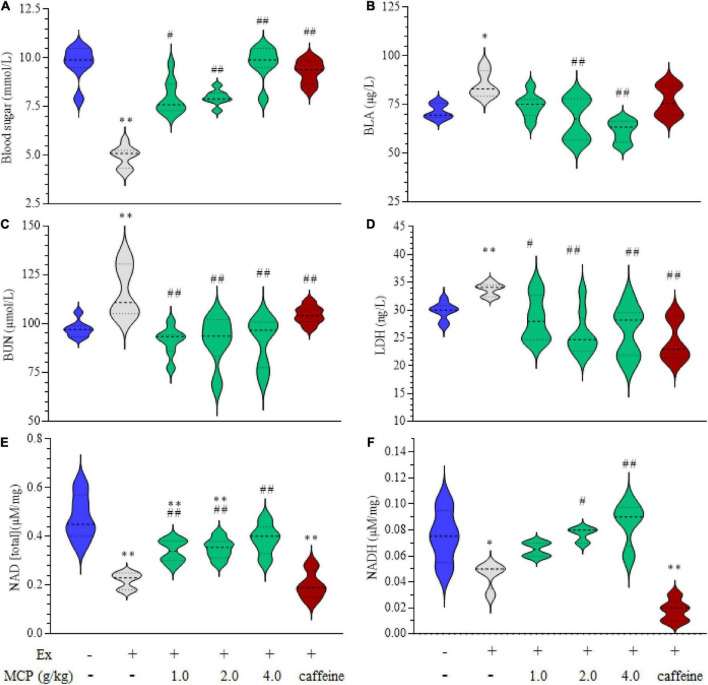
Effects of Maca compound preparation (MCP) on energetic metabolism of exercise mice. **(A)** Blood sugar. **(B)** Blood lactic acid (BLA). **(C)** Blood urea nitrogen (BUN). **(D)** Lactate dehydrogenase activity (LDH) in the serum. **(E,F)** Nicotinamide adenine dinucleotide (reduced) [NAD(H)] in the muscle. *p < 0.05, ^**^p < 0.01 vs control (Con); ^#^*p* < 0.05, ^##^*p* < 0.01 vs exercise (Ex) group.

To evaluate the energy metabolisms during fatigue, the contents of blood sugar, BLA, BUN, LDH in serum and NAD(H) in muscle were investigated in exercise mice. The occurrence of fatigue is accelerated by the decrease of blood sugar and accumulation of metabolites such as BLA, BUN and LDH, however, this situation is effectively reversed by the treatment of MCP as shown in Figures 2A–D. In addition to these metabolites, NAD(H) plays key role in the process of glycolysis process and cellular respiration, which is also an integral determining factor in fatigue (24). The levels of NAD(H) were decreased by approximately half in mice muscle after swimming, which were significantly increased by MCP-treatment compared with those of Ex group (Figures 2E,F). MCP increased energy materials in muscle, which could improve athletic capacity and enhance exercising endurance, in line with the observed results of exercise performance.

### Effect of Maca compound preparation on mice exercise-induced oxidative stress and muscle injuries

**FIGURE 3:**
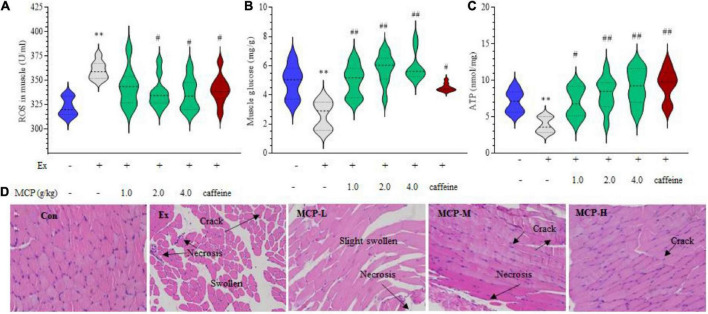
Maca compound preparation (MCP) protect muscle damage from oxidative stress in exercising mice. **(A)** Reactive oxygen species (ROS) accumulation. **(B)** Glycogen contents. **(C)** Adenosine triphosphate (ATP) generation capacity. The content of ATP was calculated as a value relative to that in Con group, which was set as 100%. ^**^p < 0.01 vs control (Con) group; ^#^*p* < 0.05, ^##^*p* < 0.01 vs exercise (Ex) group. **(D)** Hematoxylin and Eosin (H&E) stain of skeletal muscle. MCP-L, MCP-M, MCP-H represent MCP of low, moderate, and high dose levels.

The above results of changes in plasma and muscle metabolites suggested abnormal energy metabolism of exercise mice during fatigue loading. To further explore the role of MCP in resistance to acute exercise-induced peripheral fatigue, we focused on the site of muscle *in vivo*. In WFST model, the ROS levels in the left hind thigh mice muscle were significantly down-regulated by MCP supplementation compared to the Ex group (*p* < 0.05) in Figure 3A, which is parallel to the outcomes of the *in vitro* tests. In addition, MCP concentration-dependently enhanced the muscle glycogen contents (Figure 3B) and ATP level (Figure 3C), promoting energy metabolism in muscle during swimming. To intuitively observe the protective effect of MCP, H&E staining were carried out on the hind leg thigh muscle (Figure 3D). Representative images showing H&E staining on mice muscle. Like the *in vitro* studies, the intense exercise led to muscle cells swelling, necrosis or degradation with abundant cracks. Pre-treatment of MCP at moderate or high dose exhibited pronounced cytoprotective effects, and protected muscle issues from disorganization and loss of tissue structure. Based on effects of the mice physical fitness, an obvious enhancement was observed on MCP at moderate dose compared with the low dose, which was larger than that between the moderate and high doses. Thus, the moderate dose (2.0 g/kg), which was equivalent to the recommended dose in humans, was selected for subsequent experiments.

### Effect of Maca compound preparation on mice gut microbiota and correlation to the biochemical parameters

**FIGURE 4:**
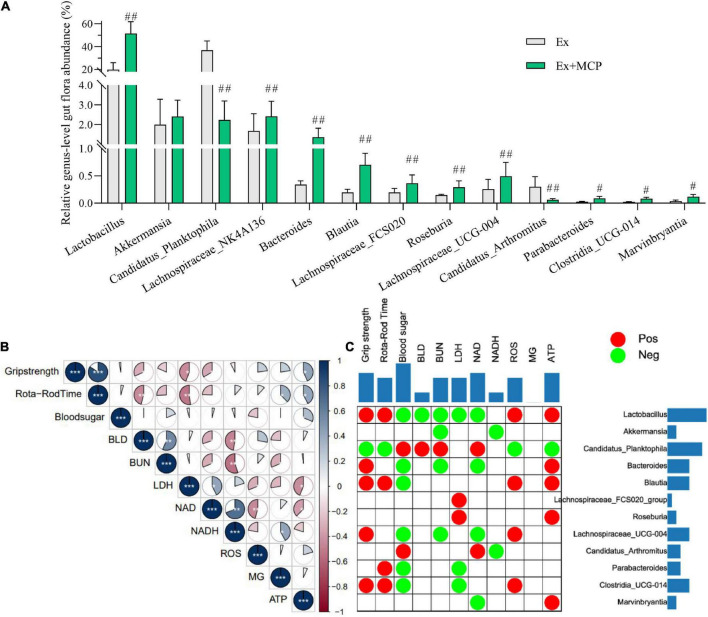
Mice gut microbiota and their correlation to the fatigue-related biochemical parameters of exercising mice. **(A)** Relative genus-level gut flora abundance; **(B)** Pearson correlation analysis among fatigue-related indicators; **(C)** Pearson correlation analysis between fatigue-related indicators and relative genus-level gut flora abundance (red plots represent *r* ≥ 0.5, and green represent *r* ≤ −0.5; the two-side blue bar represents the number of positive/negative plots in each row or column, reflecting the importance of the gut microbiota (row) or biochemical parameter (column) in the correlation grid). ^#^*p* < 0.05, ^##^*p* < 0.01 vs exercise (Ex) group.

According to the result of 16s RNA, the relative genus-level gut flora abundance was significantly different between Ex and Ex + MCP groups (Supplementary Table 1). MCP can improve resistance to fatigue by regulating the intestinal flora by increasing several primary beneficial bacteria (i.e., *Lactobacillus*, *Akkermansia* and etc.), and decreasing harmful bacteria (i.e., *Candidatus_Planktophila* and *Candidatus_Arthromitus*; Figure 4A). Notably, the relative genus-level gut flora abundance of *Lactobacillus* is increased over 2.5 folds (51.4 vs 19.8%, MCP vs Ex group), and that of *Candidatus*_*Planktophila* is decreased about 16 folds (2.2 vs 36.9%). Fatigue-related biochemical changes, as well as gut microbiota changes have been found their contributions to exercise mice performance (19, 25). In particular, fatigue generation is proved as one of the consequences of gastrointestinal imbalance, which may be associated with host metabolism and intestinal microecology (26). To further evaluate the correlations among oxidative stress and energy metabolism in serum and muscle, Pearson correlation is performed to analyze the correlations (Figure 4B). According to Pearson correlation analysis, high relevance was observed between fatigue-related biomarkers (BLD, BUN, LDH and ROS) and energy-supplying substances (blood sugar, MG and ATP), which were strongly correlated to fecal microbiota at the genus level as well (Figure 4C). Accumulating convincing evidence has shown that the gut flora plays key role in resistance to fatigue in clinic (25). According to matrix plots, the exercise performance is positively related to the relative abundance of *Lactobacillus*, *blautia*, *Clostridia*_UCG-014 ant etc. (*r* ≥ 0.5, *p* < 0.01), whereas negatively related to that of harmful bacteria (i.e., *Candidatus_Planktophila* and *Candidatus_Arthromitus*) (*r* ≤ −0.5, *p* < 0.01). The absolute values of Pearson correlation lower than 0.5 were hidden, which were accumulated and displayed in two-side. According to the cumulative value in Figure 4C, BLD is one of the most relative biomarkers indicating exercise-induced fatigue, indicating that BLD might play a key role in exercise physiological, performance, and energy utilization via microbe-host interactions (27). Meanwhile, *Lactobacillus* and *Candidatus_Planktophila* may be the most relative beneficial/harmful bacteria.

### Effect of Maca compound preparation on mice gut microbial functions

**FIGURE 5:**
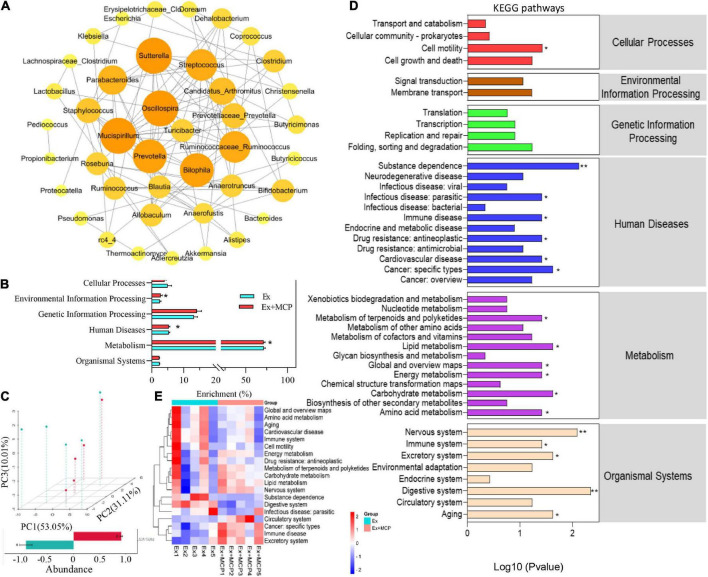
Effect of Maca compound preparation (MCP) on the mice gut microbial functions. **(A)** Correlation networks of microbe-microbe interactions regulated by MCP. Here, the lines between the circles represents a significant correlation (*p*-value < 0.05), the red represents a positive correlation, and blue represents a negative correlation. **(B)** Kyoto Encyclopedia of Genes and Genomes pathway (KEGG) of microbial (level 1). **(C)** Principal component (PCA) analysis of microbial genus levels and functional differences between Ex and Ex + MCP groups; **(D)** Heatmap of KEGG (level 2) pathways (adj. *p*-value < 0.05). **(E)** KEGG (level 2) pathways of the mice gut microbial gene functions related to cellular processes, environmental information processing, genetic information processing, human diseases, metabolism, and organismal systems. **p < 0.01.

The host-microbial and microbe-microbial interactions are often governed by the complex exchange of metabolites, which are highly associated with the metabolic capacity of hosts in health and disease (28). In Figure 5A, networks of microbe-microbe interactions revealed the regulation of MCP among microbiomes (*p* < 0.01), where *Lactobacillus* suggested that it might occupy the dominant position due to the biggest circle size. To further investigate how MCP regulates gut microbiota and metabolic processes in the fatigue, the PICRUSt2 algorithm and MicroCyc databases were employed to analyze the metabolic functions of gut microbes. By comparing with the microbial genome sequences in the database, the key pathways (L1) involved in the breakdown of MCP by microbes were most related to metabolic pathways, and significantly distinguished from Ex in the environmental information processing, human diseases pathways and metabolism (Figure 5B). In addition, the gut microbial functions were analyzed and principal component (PCA) analysis revealed striking differences between the two groups based on KEGG orthology (L2; Figure 5C). Compared with the Ex group, MCP significantly regulated 19 pathways at levels 2 (Figure 5D) and the changes in the mice individual level were shown in Figure 5E. MCP could enhance the cellular processes of cell motility. Six human diseases were downregulated by MCP, involving in substance dependence, infectious disease, immune disease, drug resistance, cardiovascular disease and cancer. Additionally, treatment with MCP significantly reduced amino acid metabolism, and global and overview maps, while MCP significantly enhanced energy metabolism, carbohydrate, lipid metabolism, metabolism of terpenoids and polyketides compared with the Ex group. MCP might be critically involved in tissue development and tissue homeostasis in aging, circulatory system, digestive system, excretory system, immune system, and nervous system. Furthermore, MCP significantly downregulated the pathways of human cytomegalovirus infection, atrazine degradation, renin angiotensin system and etc. at level 3 (Supplementary Figure 1). Thus, the nutritional modulation of gut microbiota by MCP and its interplay between intestinal microbiota and host metabolism may provide a promising insight into fatigue process and a promising avenue for some metabolic dysfunctions (29).

### Effect of Maca compound preparation on oxidative stress and energy metabolism *in vitro*

**FIGURE 6:**
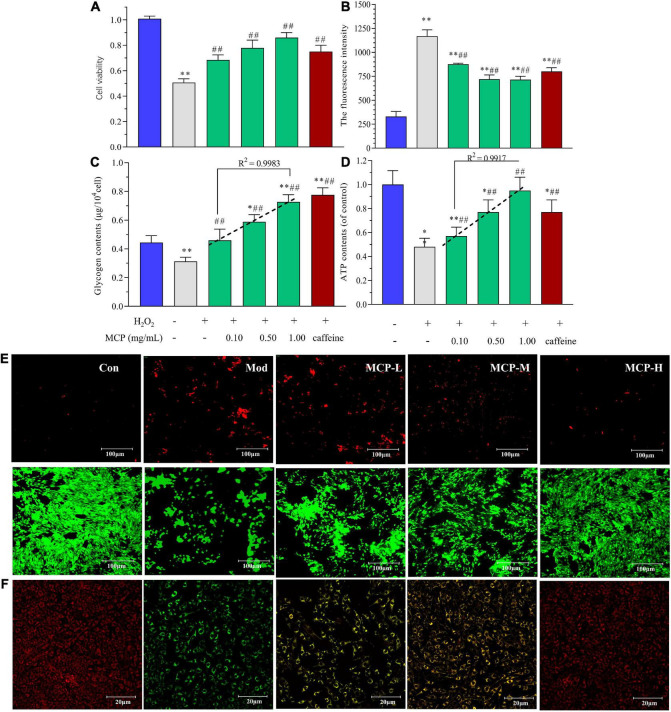
Effects of Maca compound preparation (MCP) on H_2_O_2_-induced C2C12 cells. **(A)** Cell viability of MCP on oxidative stress-induced C2C12 cells. **(B)** ROS accumulation analyzed by flow cytometry. **(C)** Glycogen contents. **(D)** ATP generation capacity. The content of ATP was calculated as a value relative to that in con group, which was set as 100%. *p < 0.05, ^**^p < 0.01 vs control (Con); ^#^*p* < 0.05, ^##^*p* < 0.01 vs model (Mod). **(E)** The Live (green)/Dead (red) assay of C2C12 treated with H_2_O_2_-induced oxidative stress. **(F)** Mitochondrial membrane potential and morphology. MCP-L, MCP-M, MCP-H represent MCP of low, moderate, and high dose levels.

Based on the above results, *in vitro* experiment was carried out to evaluate energy metabolism regulation of MCP in fatigue. According to the free radical theory, the accumulation of oxidative damage is a causal factor for muscle fatigue (30). Thus, H_2_O_2_ exposure was widely used for inducing C2C12 cell injuries to mimic oxidative damage in muscle fatigue (31, 32). Commonly, cell viability is a comprehensive index used to evaluate proliferation and cell death, indicating the potential of MCP to resist fatigue. In contrast, MCP (0.1–1.0 mg/ml) showed a marked potential for protecting skeletal muscle cells from oxidative stress injuries (Figure 6A). Oxidative stress and energy metabolism play indispensable roles in the in-tissue homeostasis and fatigue-related symptoms. The fluorescence intensity represented the amount of intracellular ROS production in C2C12 cells, which were detected by using DCFH-DA as fluorescent probe and analyzed by flow cytometry. H_2_O_2_ treatment increased ROS levels in cells, whereas MCP pre-incubation reduced H_2_O_2_-induced ROS generation (*p* < 0.01), suggesting that resistance to H_2_O_2_ injury was improved by MCP (Figure 6B). meanwhile, MCP concentration-dependently enhanced the intracellular glycogen and ATP contents, promoting energy metabolism in C2C12 cells. Under oxidative stress damage, the glycogen contents in Mod decreased significantly (^**^p < 0.01) by nearly 30%. The MCP groups showed higher contents of C2C12 glycogen and ATP levels than those of Mod groups, indicating its anti-fatigue potential on H_2_O_2_ mediated-oxidative stress on cells. Moreover, it was worth noting that MCP concentration-dependently enhanced the glycogen content (*R*^2^ = 0.9983), and ATP generation ability (*R*^2^ = 0.9917) *in vitro* (Figures 6C,D). MCP significantly conferred anti-cell necrosis ability and attenuated exercise-induced damage, as evidenced by an increase of Live/Dead assay and skeletal muscle morphology (Figure 6E). Compared with Con, the H_2_O_2_ shrunk the cells, deformed their shapes, and generated large quantities of cell debris, while MCP significantly alleviated H_2_O_2_-induced oxidative damage both in viability or cell morphology. As shown in Figure 6F, H_2_O_2_ injury impaired ΔΨm, while MCP treatment improved the stability of ΔΨm and morphology, indicating protective effects on mitochondrial functions. Thus, the *in vitro* experiments further demonstrated a substantial effect of MCP on mitochondrial function and energy metabolism.

### Exploration the potential metabolic pathways of Maca compound preparation

**FIGURE 7:**
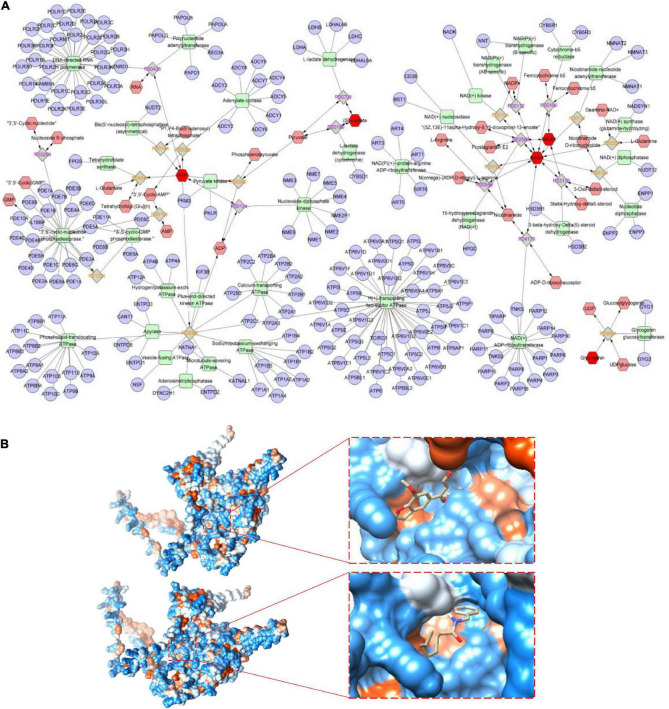
Metabolic pathways and molecular docking. **(A)** Potential metabolic network regulated by Maca compound preparation (MCP) in exercising mice. The purple circular nodes represent genes, the green square nodes represent metabolism-related enzymes, and the red hexagon nodes represent fatigue-related metabolites, involving blood lactic acid (BLA), blood sugar, glycogen, adenosine triphosphate (ATP), and nicotinamide adenine dinucleotide (reduce) [NAD(H)] determined in exercising mice. **(B)** Molecular docking of cnidilin (△G = −6.36 kcal/mol) and obacunone (△G = −6.11 kcal/mol) to the key protein of the intestine-specific expression of phosphodiesterase 9A (PDE9A).

Mitochondrial respiratory metabolism is the major source of cellular glycogen and ATP, and is associated with the proper maintenance of cellular metabolism as a whole (33). To further explore the key metabolic pathways of MCP, a joint pathway analysis was performed based on network pharmacology and metabolic pathway analyses on MetaboAnalyst platform. Eight anti-fatigue intestine-specific expressed targets (ABCG2, PDE9A, SLC6A4, CHRNA7, HNF4A and MAOA) of MCP and six metabolites (BLA, blood sugar, glycogen, ATP, NAD and NADH) were plugged into MetScape to build compound-reaction-enzyme-gene networks by matching with the potential targets obtained from MetScape (Figure 7A). The intestine-specific expression of phosphodiesterase 9A (PDE9A) was identified as the key target. Therefore, we believe that PDE9A may have a crucial effect on the efficacy of MCP for fatigue.

Several bioactive substances might play critical roles in MCP. Based on previous network pharmacology analysis (8), N-benzyl-octanamide (from Maca), vitamin E (from *Amomum villosum Lour*), cnidilin and phyllanthin (from *Angelica sinensis*), obacunone (from *Citrus reticulata Blanco*), are the active ingredients targeted on PDE9A. Figure 7B showed the visualization of the most energetically favorable binding of the six ligands into the key protein of PDE9A. As the most favorable interaction, cnidilin and obacunone showed the lowest Gibbs free energy (△G = −6.36 and −6.11 kcal/mol) and for PDE9A, suggesting that suggesting that dietary influences by MCP are most probably mediated in part by the gut microbes.

## Discussion

Fatigue were the most commonly reported systemic reactions, suggesting a link existed among fatigue-related parameters from central to peripheral (19). Intestinal dysbiosis can dysregulate inflammation of intestinal surrounding tissues as well as cognition and mood (34). In the formula, Maca could improve male sexual behavior so that it has been known as “South American ginseng” or “Plant Viagra” worldwide (5). Its extract could increase glucose uptake by inhibiting mitochondrial function (35). Natural herbal medicine with compatibility and multiple targets, is widely regarded as a new way for drugs discovery for anti-fatigue. It points out directions for future studies on the internationalization of Traditional Chinese Medicine (TCM) (36). Under complementarity principle, the prescription was expected to exert anti-fatigue activities on multiple targets with the compatibility detoxication-based TCM theory. Meanwhile, the combined extracts produced better synergistic effects than a single drug (37). Compared with conventional single-target drugs, TCM prescriptions usually contain several medicinal herbs, along with synergistic effects (multiple compounds – multiple targets – one pathway) (38, 39).

Increasing numbers of evidence suggested that gut microbiota-targeted therapy is a promising strategy to treat the chronic or metabolic disorders, which may behave as one of the mechanism for altering the pathogenesis TCM herbals (40). Currently, accumulated results have showed that *Lactobacillus* could significantly elevated the exercise performance in a dose-dependent manner and improved the fatigue-associated features correlated with better physiological adaptation (41). A recent study has revealed the impacts of gut microbiome on the host’s health, where *Lactobacillus* primarily modulated the overall microbial community structure (42). On the other hand, *Candidatus Planktophila* is an actinobacterium, which might represent pathogenic bacteria from freshwater bacterioplankton (43). *Candidatus_Arthromitus* was proved associated with depression (44). PDE9A is an intracellular cyclic guanosine monophosphate (cGMP) hydrolase, which has been exploited as one of the most promising therapeutics for treatment of metabolic diseases, such as diabetes and Alzheimer’s (45), as well as fatigue syndromes (46). In this study, the link between fatigue-related parameters and gut microbiota have been established by treatment of MCP, however, the underlying mechanisms should be further explored – the gut microbiota and the host interplay. Thus, further studies on host-microbe-drug-nutrient may probably need to answer the fundamental question of how MCP maintains intestinal microecological balance and delays generation of host fatigue (47).

In conclusion, it was demonstrated a medicinal and edible decoction – MCP, as a promising candidate to manage exercise-induced fatigue, which may be of particular importance in the case of manual workers or sub-healthy populations.

## Data availability statement

The datasets presented in this study can be found in online repositories. The names of the repository/repositories and number(s) can be found below: NCBI [BioProject: PRJNA888212 for 16S rRNA sequencing].

## Ethics statement

The animal study was reviewed and approved by the Ethics Committee of Experimental Animal Center of Jiangnan University (JN.No 20200710i0720915).

## Author contributions

HZ: conceptualization and writing – original draft preparation. RW and HH: validation and formal analysis. HQ and PD: conceptualization. All authors contributed to the article and approved the submitted version.

## Footnotes

1
http://huttenhower.sph.harvard.edu/galaxy/

2
http://swisstargetprediction.ch/index.php

3
http://swissdock.ch/

4
https://alphafold.ebi.ac.uk/

## Appendix

According to the conversion ratio between mice and humans, the daily MCP dose (2.0 g/kg) for mice can be calculated as the following formula: conversion ratio of surface area between mice and humans (10) × a daily dose of an adult (12 g)/average weight of adults (60 kg), that is: 10 × 12 (g)/60 (kg) = 2.0 g/kg.
