# Supplementary material for: Deciphering the potential role of Maca compounds prescription influencing gut microbiota in the management of exercise-induced fatigue by integrative genomic analysis
Source: Front Nutr. 2022 Oct 12;9:1004174. doi: 10.3389/fnut.2022.1004174 (PMC9597638; doi:10.3389/fnut.2022.1004174)

**Table S1 The** **relative abundance of top 20 gut bacteria in exercise mice feces**

| **No.** | **Latin name** | **Enrichment in Ex group** | **Enrichment in MCP group** |
| --- | --- | --- | --- |
| 1 | *Lactobacillus* | 19.83 % | 51.44 % |
| 2 | *Candidatus_Planktophila* | 36.89 % | 2.23 % |
| 3 | *Muribaculaceae* | 16.06 % | 13.21 % |
| 4 | *Rikenellaceae_RC9_gut_group* | 8.19 % | 3.11 % |
| 5 | *Lachnospiraceae_NK4A136_group* | 6.20 % | 4.34 % |
| 6 | *Bacillus* | 2.62 % | 4.84 % |
| 7 | *Muribaculum* | 1.99 % | 2.41 % |
| 8 | *ASF356* | 1.68 % | 2.41 % |
| 9 | *Akkermansia* | 0.30 % | 0.69 % |
| 10 | *Clostridia_UCG_014* | 1.07 % | 1.01 % |
| 11 | *Parabacteroides* | 0.34 % | 1.35 % |
| 12 | *Enterorhabdus* | 0.20 % | 0.70 % |
| 13 | *Faecalibaculum* | 1.07 % | 0.02 % |
| 14 | *Bacteroides* | 0.15 % | 0.29 % |
| 15 | *Clostridia_vadinBB60_group* | 0.20 % | 0.36 % |
| 16 | *Colidextribacter* | 0.26 % | 0.49 % |
| 17 | *Alistipes* | 0.11 % | 0.17 % |
| 18 | *Candidatus_Arthromitus* | 0.30 % | 0.06 % |
| 19 | *Lachnospiraceae_FCS020_group* | 0.20 % | 0.36 % |
| 20 | *Parvibacter* | 0.11 % | 0.17 % |

**Fig.S1 Volcano map of KEGG (level 3) pathways of the mice gut microbial functions**


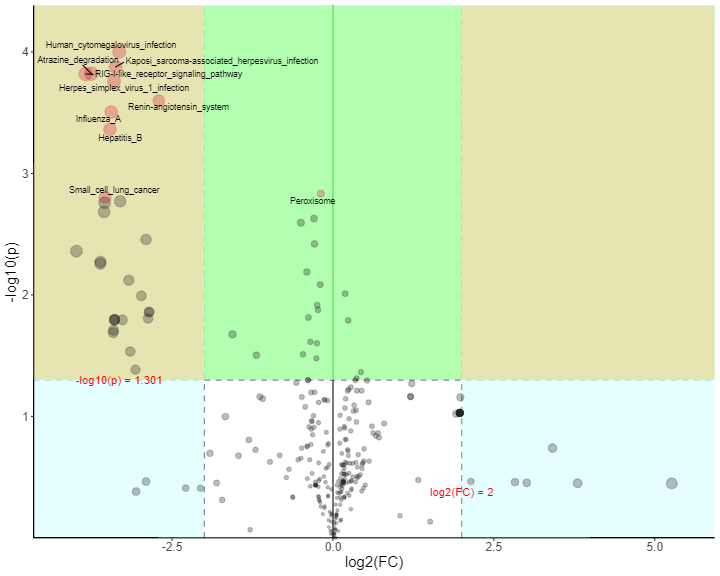

Supplement: Supplementary file 1 [file Data_Sheet_1.docx]
